# A joinpoint analysis examining trends in firearm injuries at six us trauma centers from 2016 to 2022

**DOI:** 10.1186/s40621-024-00505-5

**Published:** 2024-05-13

**Authors:** Kristin Salottolo, R. Joseph Sliter, Gary Marshall, Carlos H. Palacio Lascano, Glenda Quan, David Hamilton, Robert Madayag, Gina Berg, David Bar-Or

**Affiliations:** 1 Trauma Research Department, Swedish Medical Center, Englewood, CO USA; 2 Trauma Research Department, Medical City Plano, Plano, TX USA; 3 Trauma Research Department, Wesley Medical Center, Wichita, KS USA; 4 Trauma Services Department, Wesley Medical Center, Wichita, KS USA; 5 Trauma Services Department, Medical City Plano, Plano, TX USA; 6 Trauma Services Department, South Texas Health System, McAllen, TX USA; 7 Trauma Services Department, Swedish Medical Center, Englewood, CO USA; 8 Trauma Services Department, Penrose Hospital, Colorado Springs, CO USA; 9 Trauma Services Department, St. Anthony Hospital, Lakewood, CO USA; 10 Trauma Services Department, Lutheran Hospital, Denver, CO USA

**Keywords:** Firearm, Racial disparities, Hospitalization, Traumatic injury, Temporal trends

## Abstract

**Background:**

There is an epidemic of firearm injuries in the United States since the mid-2000s. Thus, we sought to examine whether hospitalization from firearm injuries have increased over time, and to examine temporal changes in patient demographics, firearm injury intent, and injury severity.

**Methods:**

This was a multicenter, retrospective, observational cohort study of patients hospitalized with a traumatic injury to six US level I trauma centers between 1/1/2016 and 6/30/2022. ICD-10-CM cause codes were used to identify and describe firearm injuries. Temporal trends were compared for demographics (age, sex, race, insured status), intent (assault, unintentional, self-harm, legal intervention, and undetermined), and severity (death, ICU admission, severe injury (injury severity score ≥ 16), receipt of blood transfusion, mechanical ventilation, and hospital and ICU LOS (days). Temporal trends were examined over 13 six-month intervals (H1, January–June; H2, July–December) using joinpoint regression and reported as semi-annual percent change (SPC); significance was *p* < 0.05.

**Results:**

Firearm injuries accounted for 2.6% (1908 of 72,474) of trauma hospitalizations. The rate of firearm injuries initially declined from 2016-H1 to 2018-H2 (SPC = − 4.0%, *p* = 0.002), followed by increased rates from 2018-H2 to 2020-H1 (SPC = 9.0%, *p* = 0.005), before stabilizing from 2020-H1 to 2022-H1 (0.5%, *p* = 0.73). NH black patients had the greatest hospitalization rate from firearm injuries (14.0%) and were the only group to demonstrate a temporal increase (SPC = 6.3%, *p* < 0.001). The proportion of uninsured patients increased (SPC = 2.3%, *p* = 0.02) but there were no temporal changes by age or sex. ICU admission rates declined (SPC = − 2.2%, *p* < 0.001), but ICU LOS increased (SPC = 2.8%, *p* = 0.04). There were no significant changes over time in rates of death (SPC = 0.3%), severe injury (SPC = 1.6%), blood transfusion (SPC = 0.6%), and mechanical ventilation (SPC = 0.6%). When examined by intent, self-harm injuries declined over time (SPC = − 4.1%, *p* < 0.001), assaults declined through 2019-H2 (SPC = − 5.6%, *p* = 0.01) before increasing through 2022-H1 (SPC = 6.5%, *p* = 0.01), while undetermined injuries increased through 2019-H1 (SPC = 24.1%, *p* = 0.01) then stabilized (SPC = − 4.5%, *p* = 0.39); there were no temporal changes in unintentional injuries or legal intervention.

**Conclusions:**

Hospitalizations from firearm injuries are increasing following a period of declines, driven by increases among NH Black patients. Trauma systems need to consider these changing trends to best address the needs of the injured population.

**Supplementary Information:**

The online version contains supplementary material available at 10.1186/s40621-024-00505-5.

## Background

There is an epidemic of firearm injuries in the United States (US) that impacts all ages, sexes, and races, but disproportionally affects males and racial/ethnic minorities (Fontanarosa and Bibbins-Domingo 2022; Mueller et al. 2023). More than 100,000 persons in the US suffer a firearm injury each year (Centers for Disease Control and Prevention 2023a). The US leads developed nations in firearm mortality, with nearly 49,000 firearm related deaths in 2021, up 30% from 38,000 deaths in 2016 (Centers for Disease Control and Prevention 2023b). Studies have reported a temporal increase in firearm related homicides and suicides over time (Wintemute 2015), and a spike during 2020, coinciding with the COVID-19 pandemic (Donnelly et al. 2023; McGraw et al. 2022). The largest temporal increases in firearm related homicides and suicides occurred among non-Hispanic (NH) black and other minority groups (Kegler et al. 2022).

Much is known about firearm mortality due to several available US national repositories including the Centers for Disease Control and Prevention (CDC) WISQARS and National Violent Death Reporting System, and the CDC WONDER National Vital Statistics System (Centers for Disease Control and Prevention 2023a, b). However, nearly 80% of victims of firearm violence survive their injury and there is no adequate national repository to track nonfatal firearm injuries (National Opinion Research Center (NORC) at the University of Chicago 2020) Studies examining temporal changes in firearm related injuries across all ages report disparate findings, with some studies suggesting increases (Livingston et al. 2014) and others demonstrating no change (Cook et al. 2017; Davoudi and Woodworth 2023) or declines in hospitalization (Gross et al. 2017) due to firearm injuries. At a national level, firearm related emergency department (ED) visits have remained steady, but the patterns of ED visits due to firearms are changing with declines in assaults and increases in unintentional firearm injuries (Kalesan et al. 2021).

However, most publications reporting on firearm injuries and hospitalizations present trends through 2016 (Cook et al. 2017; Gross et al. 2017; Kalesan et al. 2021, 2018; Gani et al. 2017; Smart et al. 2021). It is likely these data are not representative of present trends in firearm violence because there has been a nearly 30% increase in firearm mortality from 2016 to 2021 (Centers for Disease Control and Prevention 2023a), an increase in gun ownership from 39 to 45% from 2016 to 2021 (The Gallup Organization Guns 2024; Percentage of households in the United States owning one or more firearms from 1972 to 1972 2024), and an increase in permitless carry laws from 10 States in 2016 to 23 States in 2022 (Wikipedia Contributors 2024). Additionally, since 2016 there have been noteworthy events such as the COVID pandemic, demographic changes in the US with declines in the non-Hispanic (NH) white population (Census.gov. 2024), and the end of the federal funding freeze on gun violence research after more than 20 years (Rostron 2018).

The purpose of this study was to examine changes in firearm-related hospitalizations since 2016. Contemporaneous data-informed research is needed to understand who is affected by firearms, how frequently it occurs, and patterns of change so that we can properly address and treat firearm injuries. The specific aims were to determine the incidence of firearm injury hospitalizations since 2016 as well as temporal changes, demographic trends in firearm injury hospitalizations, and trends in firearm injury hospitalizations by intent and severity.

## Methods

This was a multicenter, retrospective, observational cohort study of six non-academic U.S. trauma centers that form a collaborative network for trauma research. The study facilities are level I trauma centers in the Central and Mountain West regions of the United States; level I centers provide the highest level of care for trauma patients. The facilities are located in smaller cities (Penrose Hospital, Colorado Springs, CO; STHS-McAllen Hospital, McAllen TX; Wesley Hospital, Wichita, KS) or in suburban areas near Denver, CO and Dallas, TX (Swedish Medical Center, Englewood CO; St. Anthony Hospital, Lakewood CO; Medical City Plano, Plano TX).

The trauma registry was used to identify adult (age ≥ 18) trauma patients who arrived between 1/1/2016 and 6/30/2022. Patients whose race was unknown or not documented were excluded, which was just over 3% of patients.

In order to be included in the trauma registry of participating hospitals, patients needed to have been admitted to the hospital or observed, died during transit, or died in the ED. Patients who died at the scene or patients who were discharged from the ED were excluded. The trauma registry includes data from time of injury through discharge (or death); there were no post-hospitalization data available.

The 10th revision of the International Statistical Classification of Diseases, Clinical Modification (ICD-10-CM) external cause code was used to identify firearm-related injuries, intent of firearm injury, and type of firearm used. Firearm injury intent was defined as assault (including homicide), unintentional (accidental) discharge, self-harm (suicide/attempt), legal intervention (law enforcement), and undetermined intent. If the ICD-10-CM external code was missing the trauma registrar-assigned cause code was used (*n* = 10, 0.5% with firearm injury). Type of firearm was defined as handgun (e.g., pistol), longarm (e.g., shotguns, rifles, and other long barreled firearms), unspecified firearm discharge, and other firearm discharge.

Demographics were examined as age (18–30 vs. > 30 years, based on the median age of 30 years), sex, insurance status (uninsured/self-pay vs. insured) and race: Hispanic, NH White, NH Black, NH Asian American or Pacific Islander or American Indian / Alaska Native (AAPI /AIAN), and NH-other (patients that self-identified as “other race”); one facility did not differentiate race and ethnicity, and thus Hispanic ethnicity was coded as a race for our analysis. At the other five facilities, Hispanic patients who also self-identified as either Black or AAPI/AIAN were categorized as Hispanic.

Severity and resource utilization was examined with the injury severity score (ISS; < 16 vs. ≥ 16), receipt of a blood transfusion (yes/no), mechanical ventilation (yes/no), intensive care unit (ICU) admission (yes/no), ICU length of stay (LOS, days), hospital LOS, and death (vs. discharged alive).

### Statistical analysis

SAS® version 9.4 was used to summarize data. Joinpoint regression analyses were used to analyze temporal changes in firearm injuries using the National Cancer Institute (NCI) joinpoint software program version 5.0.2 (Joinpoint Regression Program, Version 5.0.2 2023), using the methods proposed by Kim et al. (2022). A joinpoint regression model segments time series data into groups of data points with similar linear trends to identify inflection points (i.e., joinpoints). The time series used semi-annual (every 6 months, where H1 is January through June and H2 is July through December) averages and standard errors in firearm hospitalization rates to report the semi-annual percentage change (SPC). A linear model with zero joinpoints was initially fit and additional joinpoints were added when the slope of the line between joinpoints was significantly different from zero (*p* < 0.05 based on the SPC compared to zero). A stable or non-significant trend was defined based on a *p* ≥ 0.05 when comparing the SPC to zero. The maximum number of joinpoints for our dataset was 3 based on 13 data points in our series. Joinpoint models were used to examine overall changes in firearm injuries over time as well as temporal changes by demographic characteristics (age, sex, race, insurance status), firearm injury intent and firearm type, and severity and resource use (ISS, blood transfusion, mechanical ventilation, ICU admission, ICU and hospital LOS, and death).

Due to the complexity of interpreting the joinpoint analysis, we also performed a supplementary analysis of linear, annual trends in firearm injury hospitalization, intent, demographics, and severity characteristics in SAS® using Cochran-Armitage trend tests (Supplementary Table 1).

## Results

### Firearm-related injuries, overall and by hospital

There were 72,474 trauma patients identified and 1908 (2.6%) were injured by firearm. Compared to patients injured by other means, patients injured by firearm were more likely to be younger, male, uninsured, and were less likely to be white and more likely to be racial and ethnic minority groups; they also had worse injuries including greater than two-fold rates of severe injury (ISS ≥ 16), mechanical ventilation, and blood transfusions, and had significantly greater mortality compared to patients injured by non-firearm means (Table 1).

**Table 1:** Comparison of traumatic injuries by firearm, using trauma registry data at 6 US level I trauma centers, 1/1/2016–6/30/2022

Variable	Firearm injury (*n* = 1908)	Non-firearm injury (*n* = 70,566)	*p* value
Demographics			
NH white	942 (49.4)	52,348 (74.2)	** < 0.001**
Hispanic	395 (20.7)	11,328 (16.1)	** < 0.001**
NH black	463 (24.3)	2857 (4.1)	** < 0.001**
NH AAPI/AIAN	30 (1.6)	1727 (2.5)	**0.01**
NH other	78 (4.1)	2306 (3.3)	**0.05**
Age 18–30	873 (45.8)	10,406 (14.8)	** < 0.001**
Female sex	279 (14.6)	31,547 (44.7)	** < 0.001**
Uninsured/self-pay	853 (44.7)	8088 (11.5)	** < 0.001**
Severity and resource utilization			
ISS ≥ 16	574 (30.1)	11,466 (16.3)	** < 0.001**
Respiratory ventilation	485 (25.5)	4836 (6.9)	** < 0.001**
Blood transfusion	387 (20.3)	5640 (8.0)	** < 0.001**
ICU admission	847 (44.4)	22,860 (32.4)	** < 0.001**
Median hospital LOS	3 (1–7)	4 (2–6)	** < 0.001**
Median ICU LOS	3 (2–5)	3 (2–5)	0.09
Mortality	283 (14.8)	1944 (2.8)	** < 0.001**

Data are presented as *n* (%) or median (interquartile range). Bolding denotes significance, *p* < 0.05

*NH* non-hispanic, *AAPI/AIAN* Asian American Pacific Islander, *AIAN* American Indian Alaska Native, *ISS* injury severity score, *ICU* intensive care unit, *LOS* length of stay, *ICU LOS* calculated for those admitted to the ICU

**Fig. 1:**
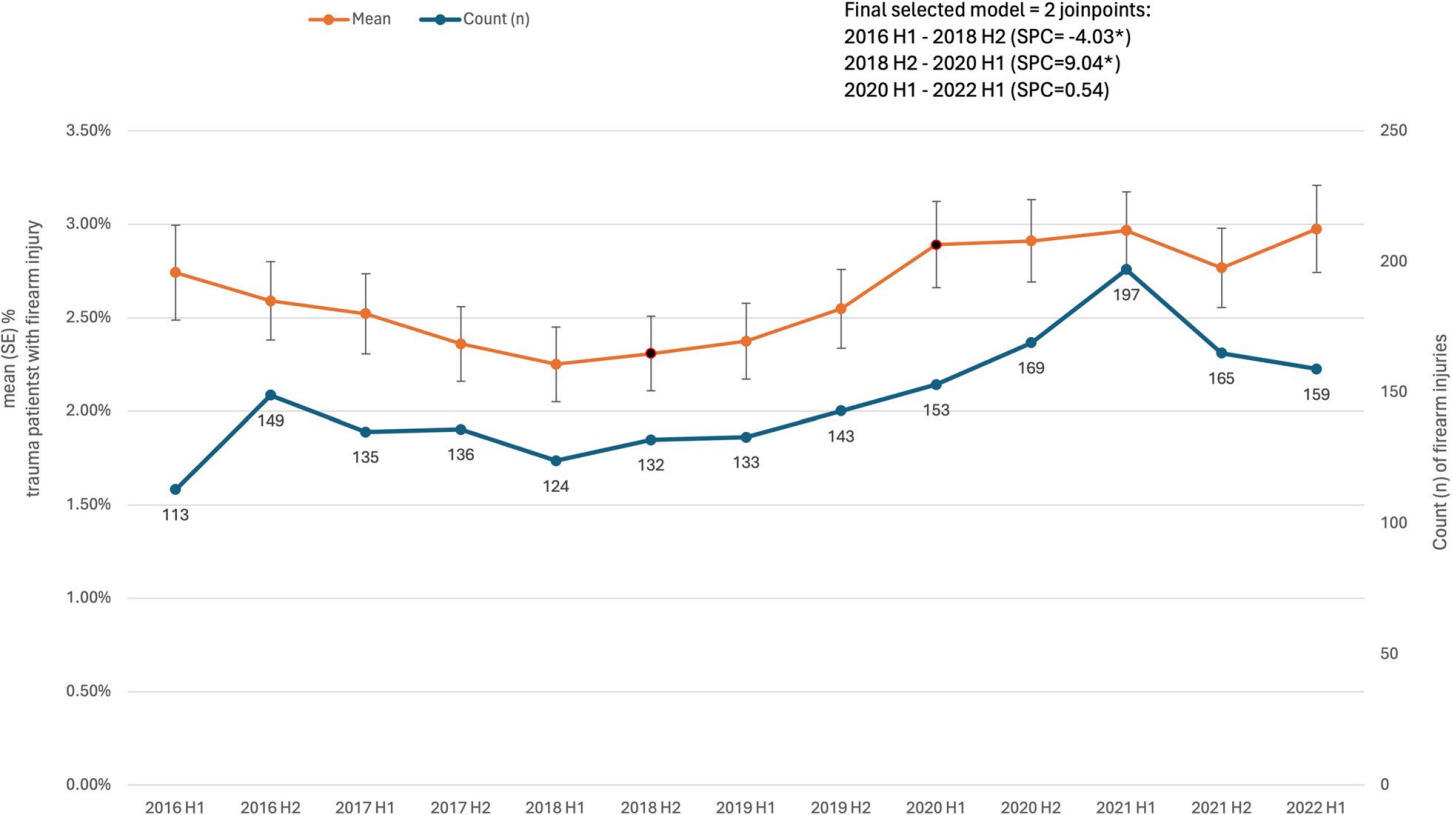
Semi-annual percent change (SPC) of trauma admissions with firearm injuries. Legend: Data from registries of 6 US level I trauma centers, 1/1/2016–6/30/2022. H1, January–June; H2, July–December. * denotes *p* < 0.05

The final model examining changes in firearm injuries over time included 2 joinpoints: the rate of firearm injuries initially declined between the start of 2016 to the second half of 2018 (SPC = − 4.0%, *p* = 0.002), followed by increased rates from the second half of 2018 to the first half of 2020 (SPC = 9.0%, *p* = 0.005), before stabilizing through the first half of 2022 (0.5%, *p* = 0.73), Fig. 1. The absolute number of firearm hospitalizations also increased over time (Fig. 1).

There were some differences across facilities in trends of firearm injuries over time. For five of six hospitals, there was a non-significant linear change in firearm injuries over time (SPCs = 0.7, 1.3, 1.6, 4.0, 5.5%). For the sixth facility, there was no initial change in firearm injuries from 2016 through 2019 (SPC = 0.4%), followed by an increase from 2019-H2 to 2021-H1 (SPC = 17.6%, *p* < 0.001), then a decline from 2021-H1 to 2022-H1 (SPC = − 11.9%, *p* < 0.001).

### Trends in firearm type and intent

Approximately half (49.7%) of firearm injuries involved a handgun, 9.2% involved a longarm, 41.1% had an ‘unspecified’ firearm discharge, and 2.2% had ‘other’ type of firearm discharge. When examined by type, there was a significant increase in firearm injuries involving an unspecified firearm (SPC = 4.9%, *p* < 0.001), a significant decline in firearm injuries involving a longarm (SPC = − 8.5%, *p* = 0.04), and no change over time in firearm injuries involving a handgun (SPC = − 2.2%, *p* = 0.09).

**Fig. 2:**
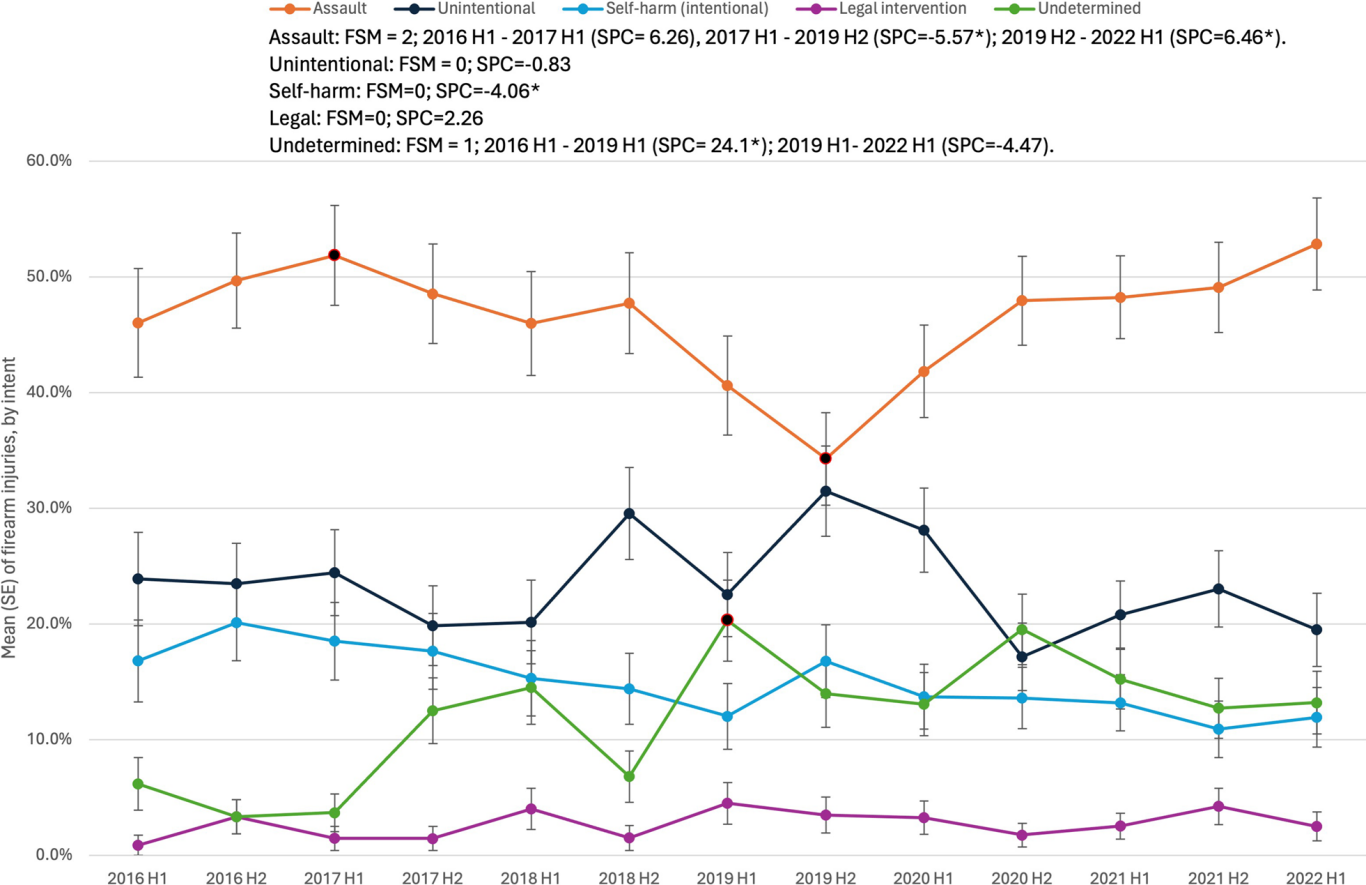
Semi-annual percent change (SPC) by intent for patients with firearm injuries. Legend: Data from trauma registries at 6 US level I trauma centers, 1/1/2016–6/30/2022. H1, January–June; H2, July–December. FSM = Final selected model (number of joinpoints). * denotes *p* < 0.05

Nearly half (46.8%) of firearm injuries were due to assault, followed by unintentional discharge (23.3%), self-harm (14.9%), undetermined intent (12.3%), and legal intervention (2.7%). Self-harm injuries declined over time (SPC = − 4.1%, *p* < 0.001), Fig. 2. There were no temporal changes in unintentional injuries (SPC = − 0.8%, *p* = 0.66) or legal intervention (SPC = 2.3%, *p* = 0.50). Undetermined injuries increased between 2016 and the first half of 2019 (SPC = 24.1%, *p* = 0.01) then stabilized (SPC = − 4.5%, *p* = 0.39). The model examining assaults demonstrated two joinpoints: there was an initial increase that was not significant from 2016 to the first half of 2017 (SPC = 6.3%, *p* = 0.16), then assaults declined from the second half of 2017 to the second half of 2019 (SPC = − 5.6%, *p* = 0.01) before increasing in 2020 to 2022 (SPC = 6.5%, *p* = 0.01).

### Demographic trends of patients injured by firearm

NH white patients comprised half (49.4%) of firearm hospitalizations, 24.3% were NH black, 20.7% were Hispanic, 1.6% were AAPI/AIAN, and 4.1% were NH other; four patients self-identified as Hispanic and Black or AAPI/AIAN. Firearm injuries accounted for 14.0% of all hospitalizations for traumatic injury for NH black patients, whereas firearm injury accounted for between 1.7 and 3.4% of trauma admissions for all other groups.

**Fig. 3:**
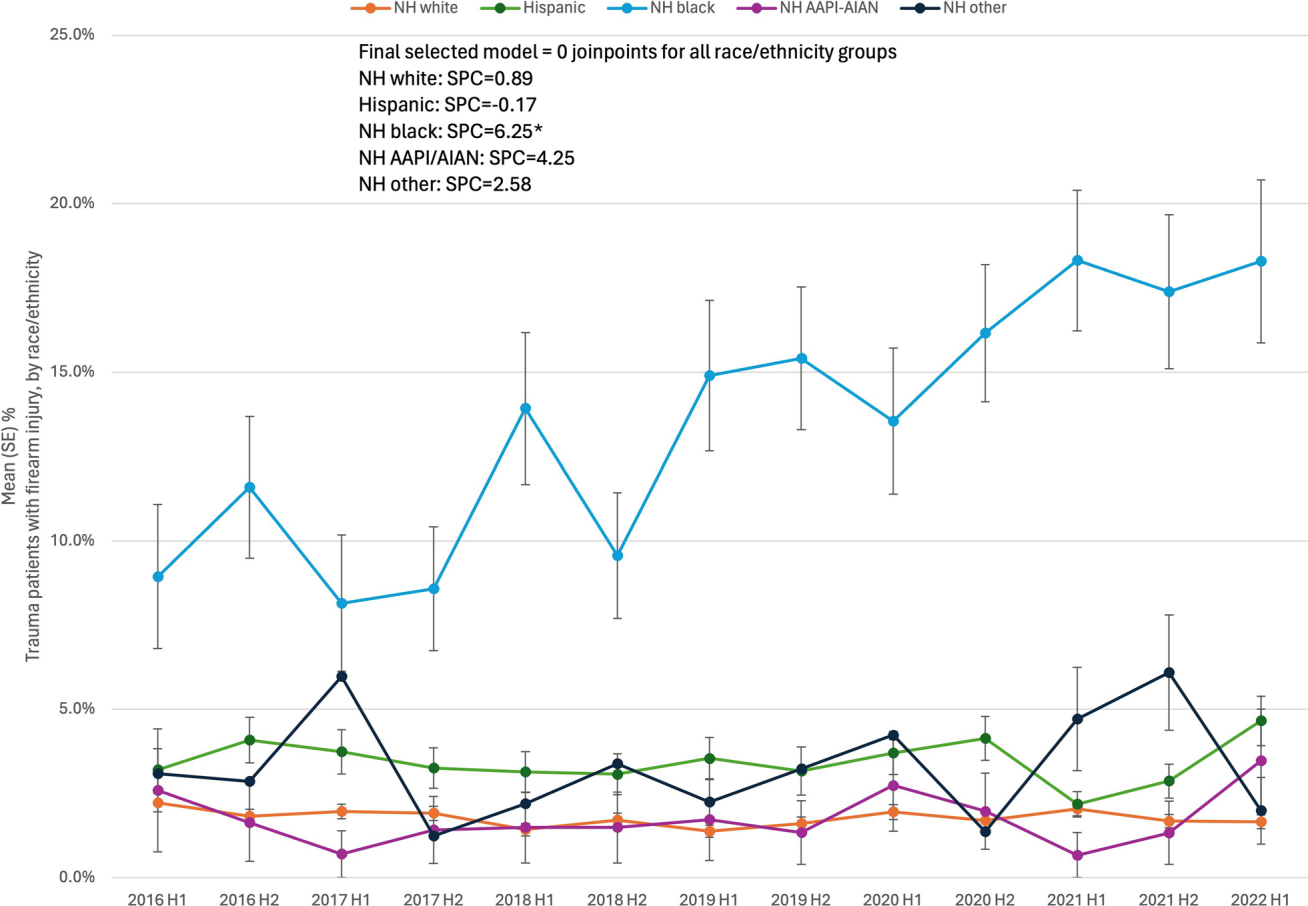
Semi-annual percent change (SPC) of patients with firearm injuries, by race/ethnicity. Legend: Data from trauma registries at 6 US level I trauma centers, 1/1/2016–6/30/2022. H1, January–June; H2, July – December. * denotes *p* < 0.05

The joinpoint analysis by race produced linear trends (0 joinpoints). NH black patients were the only group to have a significant change in firearm injuries, increasing 6.3% on a bi-annual basis (SPC = 6.3%, *p* < 0.001). There were no changes for any other race/ethnicity group (Fig. 3). The proportion of uninsured/self-pay patients also significantly increased over time (SPC = 2.3%, *p* = 0.02), while there were no temporal changes in the proportion of firearm victims that were 18–30 years old (SPC = 0.7%, *p* = 0.39) or who were female (SPC = − 0.5%, *p* = 0.73).

### Trends in severity and resource use of patients injured by firearm

**Fig. 4:**
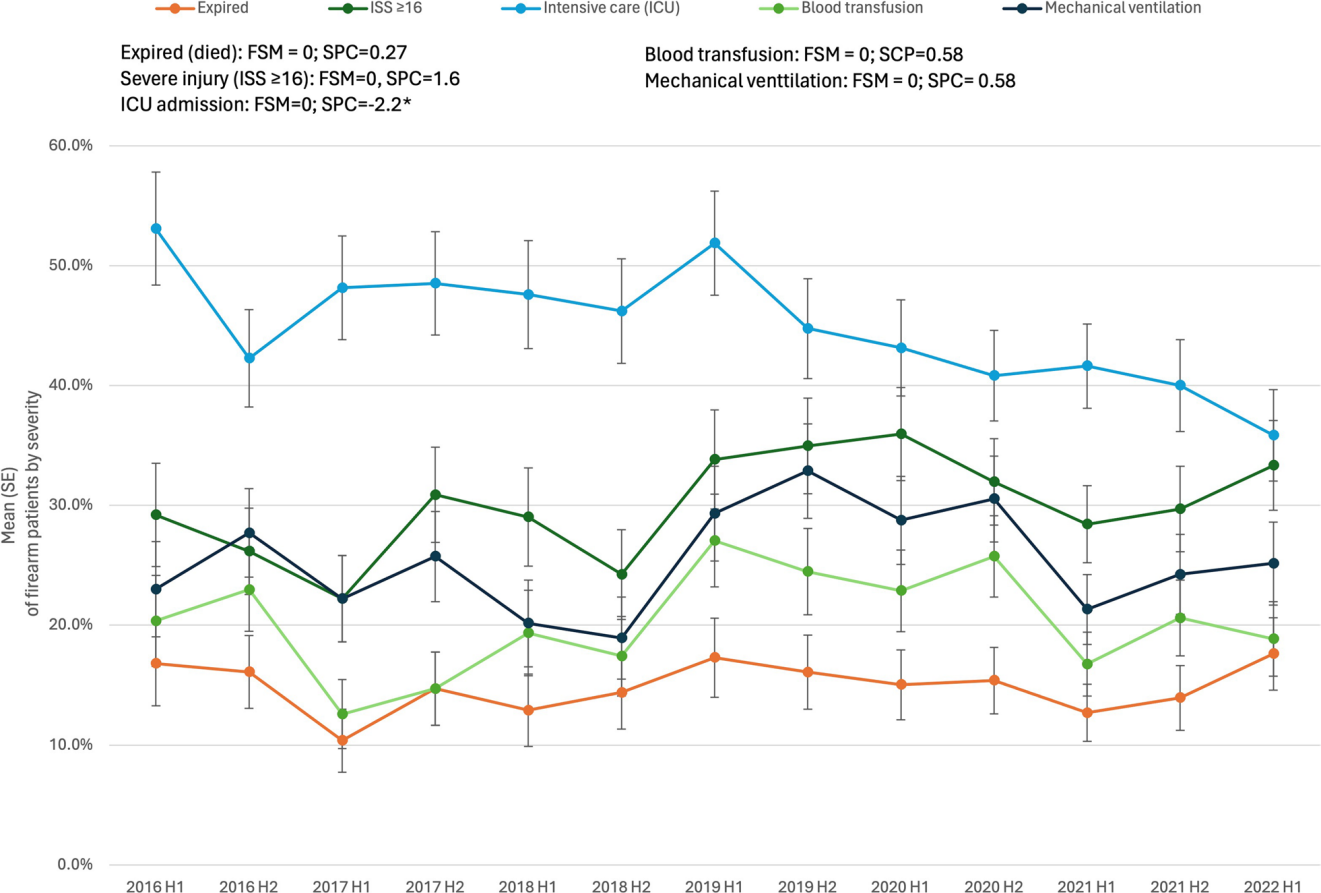
Semi-annual percent change (SPC) by severity and resource utilization for patients with firearm injuries. Legend: Data obtained from trauma registries at 6 US level I trauma centers from 1/1/2016 to 6/30/2022. H1, January–June; H2, July–December. FSM = Final selected model. * denotes *p* < 0.05

The joinpoint analysis by severity produced linear trends (0 joinpoints), Fig. 4. ICU admission rates significantly declined (SPC = − 2.2%, *p* < 0.001). There were no significant changes over time in rates of severe injury (SPC = 1.6%, *p* = 0.22), blood transfusion (SPC = 0.6%, *p* = 0.68), and mechanical ventilation (SPC = 0.6%, *p* = 0.72). There were also no changes in mortality over time (SPC = 0.3%, *p* = 0.77). The majority (50.9%) of firearm deaths were from self-harm, 31.3% were homicide/assault, 5.0% of deaths involved legal intervention, 3.2% of deaths were unintentional, and 9.6% of deaths from firearm were undetermined. Most (66.1%) patients died on the day of admission while 8.5% died en route or in the ED and 25.4% died later in the hospitalization.

**Fig. 5:**
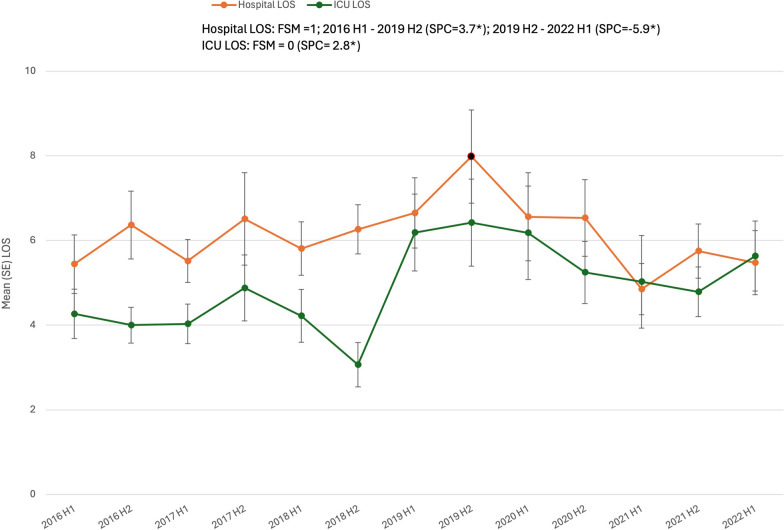
Semi-annual percent change (SPC) of hospital and ICU LOS (days) for patients with firearm injuries. Legend: Data obtained from trauma registries at 6 US level I trauma centers from 1/1/2016 to 6/30/2022. H1, January–June; H2, July–December. FSM = Final selected model. * denotes *p* < 0.05. ICU LOS calculated for those admitted to the ICU

The mean number of days in the ICU LOS increased by 2.8% bi-annually (SPC = 2.8%, *p* = 0.04), whereas hospital LOS initially increased from 2016 through 2019 (SPC = 3.7%, *p* = 0.03), then began declining in 2020 (SPC = − 5.9%, *p* = 0.03), Fig. 5.

## Discussion

This study demonstrated significant changes in hospitalization from firearm-related injuries over time. The rate of firearm injuries initially declined from 2.7 to 2.2% of all trauma hospitalizations between 2016 and the first half of 2018, significantly increasing to approximately 3% through the first half of 2020, and leveling out at 3% through the first half of 2022. This significant rise in firearm hospitalizations occurred prior to the COVID-19 period, and only one of the six hospitals in our study had a notable spike in 2020 and the first half of 2021 that coincided with the pandemic. This study also showed that firearm injuries are increasing in suburban and semi-urban settings in the U.S., reflecting national trends and bringing to light that firearm hospitalizations do not solely impact academic medical centers in large metropolitan areas. These results suggest that hospitals and trauma systems should plan and prepare for increased firearm admissions and the accompanying high resource utilization.

Racial disparities persisted during the study period. While this finding is not surprising, what is concerning is that the disparities appeared to strengthen during this recent 6.5-year time frame. Specifically, there was a high prevalence of firearm injuries among NH black patients, and this was the only group to see a steady increase in firearm hospitalizations over time, doubling from 9% of all trauma admissions in 2016 to 18% in 2022. Rates of firearm injuries for NH black patients also peaked in the second half of 2020 through the first half of 2021, overlapping with the COVID pandemic and Black Lives Matter protests following the murder of George Floyd.

The proportion of patients who were uninsured (or self-pay) increased over time, which hospital systems should be aware of and plan for. Hospitalization costs for firearm injuries are over $1 billion annually which is nearly triple that of non-firearm related care (U.S. Government Accountability Office 2021), with total hospital charges for firearm injuries ranging from $116,000 to $214,000 per patient in 2016–2019 (Silver et al. 2023).

Firearm injuries of undetermined intent significantly increased from 2016 through the first half of 2019 before trending lower, whereas firearm injuries due to assault followed the opposite pattern, significantly decreasing between 2017 through 2019 before increasing in 2020–2022. An analysis comparing ICD-9 coded firearm injuries to researcher-adjudicated intent at three level I trauma centers reported undetermined firearm injuries most closely resembled assaults (Miller et al. 2022). One interpretation of these trends we observed is that assaulted patients may not be disclosing their attack, potentially for fear of retribution, which may explain the increase in undetermined injuries and parallel decrease in assaults between 2016 and 2019 that warrants further study.

Self-inflicted firearm injuries decreased over the study period, from 17 to 12% of firearm injuries. Unintentional firearm injuries remained stable during the study period and represented nearly one-fourth of all admissions due to firearms—a substantial amount given this population excluded children and teens who are frequently the victims of unintentional firearm injuries (Wilson et al. 2023). There may be opportunities for directing gun owners to firearm safety education or implementing changes to policies requiring firearm safety education. In the states studied, both Texas and Kansas passed constitutional carry laws during the study period, removing the requirement for taking a concealed carry class prior to obtaining a permit. Prior research suggests that states that no longer required training for concealed carry weapons had a 32% increase in gun assaults (Doucette et al. 2023). Further, in person education opportunities for new firearm owners may have been limited in the setting of the COVID pandemic. This combination could increase the risk of unintentional injuries, though more specific research would be needed to assess this risk. Prior evidence-based reviews demonstrate gun locks and other safe storage methods prevent unintentional firearm injuries (Violano et al. 2018). One opportunity for further research would be to examine unintentional injuries by specific activity, to determine whether these injuries are occurring during firearm manipulation, cleaning, training, or inadvertent discharge, which would provide better insight into specific education that could address these injuries.

Severity and resource utilization from firearm injuries was high, with 44% admitted to the ICU, 26% ventilated and one in five patients requiring a blood transfusion. However, this study did not identify temporal changes in severity from firearm injuries, including mortality. Prior research suggests firearm death rates have remained relatively stable during the twenty-first century (2000–2012), following a 31% decline during the 1990s (Fowler et al. 2015).

On the contrary, we identified a significant decline in ICU admission rates and an increase in ICU admission LOS (days). One possible explanation for these findings, without a change in other markers of severity, is that the Brain Injury Guidelines (BIG) were implemented at varying times across facilities during the study period (Joseph et al. 2014). The BIG protocol grades patients with traumatic brain injury (TBI) by severity and only the most severe patients (BIG 3) require ICU admission; historically, treatment of TBI included ICU admission to monitor the patient closely for clinical deterioration. We also found an increase in hospital LOS from 2016 through 2019, followed by a significant decline in hospital LOS in 2020–2022. This latter finding may be related to the COVID-19 pandemic, as prior studies have shown a decreased LOS in trauma patients during 2020 as a result of the pandemic (Berg et al. 2021; Yeates et al. 2022).

The primary study limitation is that trauma registry data underestimates firearm injuries, especially because between 53 and 59% of firearm victims deaths occur at the scene (Agoubi et al. 2023; Sauaia et al. 2016), and upwards of 88% of suicide victims die at the scene (Kaufman et al. 2021). The trauma registry also excludes patients that were routinely discharged from the ED. In one study conducted at a level I trauma center, 19% of firearm victims were discharged from the ED, most with very peripheral or tangential gunshot wounds (Livingston et al. 2014). Across all EDs including non-trauma centers approximately 43% of firearm victims are treated and released (Fowler et al. 2015). Additionally, patients who do not seek medical care are also excluded, but without a national repository the estimates are not known.

Generalizability is another study limitation, as the study was conducted at level 1 trauma centers that treat a higher volume of serious firearm injuries requiring hospitalization compared to than lower level or non-trauma centers (Coupet et al. 2019; Hatfield et al. 2024). Moreover, three of six study sites are located in Colorado which has one of the lowest rates of firearm injuries (Smart et al. 2021). We also excluded children; in 2021, firearm injuries were the leading cause of death among US children and adolescents (Roberts et al. 2023) and the burden of pediatric firearm death has disproportionately affected communities of color (Olufajo et al. 2020). Prior research has shown that the incidence of firearm injuries is increasing in children < 18 years of age (Fraser Doh et al. 2023), similar to what we found in our adult population. A final limitation is a potential bias in ICD10 coding that has been demonstrated to overestimate unintentional injuries (Miller et al. 2022; Barber et al. 2021). The Center for Medicaid and Medicare Services codebook states “Undetermined intent is only for use when there is specific documentation in the record that the intent of the injury cannot be determined. If no such documentation is present, code to accidental (unintentional).” Our data did not show an increase in unintentional injuries but rather an increase in undetermined intent injuries, where the cause of the injury could not be determined. Another area of study is to evaluate whether clinician training on firearm counseling and intervention practices, or greater implementation of hospital-based violence intervention programs, may improve documentation of intent (among other benefits). Medical professionals are in a unique position to apply evidence-based strategies to address firearm violence (Betz et al. 2022; Cunningham et al. 2009), but prior studies have shown that only 24% of level I trauma centers conduct firearm screening and intervention (Bulger et al. 2022). Moreover, the hospitals in our study, being ACS verified level I trauma centers, have a responsibility to explore ways to mitigate firearm violence through injury prevention efforts.

## Conclusion

Our findings provide a contemporary overview of the change in firearm hospitalizations, demonstrating a period of initial declines followed by an increase beginning in 2018, prior to the COVID-19 pandemic. The increase in firearm injuries was driven by persisting racial disparities in firearm violence, with a significant, steady increase in firearm injuries for NH black patients. Hospitals, trauma systems and injury prevention programs will need to attend to these changing trends to best address the needs of the injured population.

### Supplementary Information

Additional file 1.

## Data Availability

The datasets used and/or analyzed during the current study are available from the corresponding author on reasonable request.

## Abbreviations

AAPI: Asian American Pacific Islander
AIAN: American Indian Alaska Native
BIG: Brain injury guidelines
CDC: Centers for Disease Control and Prevention
COVID-19: Coronavirus disease 2019
ED: Emergency department
EMS: Emergency medical services
ICD: International Statistical Classification of Diseases
ICU: Intensive care unit
ISS: Injury severity score
LOS: Length of stay
NH: Non-Hispanic
TBI: Traumatic brain injury
US: United States
