# Supplementary material for: A joinpoint analysis examining trends in firearm injuries at six us trauma centers from 2016 to 2022
Source: Inj Epidemiol. 2024 May 13;11:18. doi: 10.1186/s40621-024-00505-5 (PMC11092259; doi:10.1186/s40621-024-00505-5)
Supplement: Supplementary file 1 — Additional file 1. [file 40621_2024_505_MOESM1_ESM.docx]

**Supplementary Table 1. Linear annual trends in demographics, intent, and severity for firearm injury hospitalizations**

| Characteristic | 2016  (n=262) | | | 2017  (n=271) | | | 2018  (n=256) | | | 2019  (n=276) | | | 2020  (n=322) | | | 2021 (n=362) | | | H1 2022 (n=159) | | | Trend  p value |  |  |
| --- | --- | --- | --- | --- | --- | --- | --- | --- | --- | --- | --- | --- | --- | --- | --- | --- | --- | --- | --- | --- | --- | --- | --- | --- |
| *Demographics* |  | | |  | | |  | | |  | | |  | | |  | | |  | | |  |  |  |
| Age 18-30 years | 121 (46.2) | | | 110 (40.6) | | | 116 (45.3) | | | 120 (43.5) | | | 166 (51.6) | | | 176 (48.6) | | | 64 (40.3) | | | 0.35 |  |  |
| Male sex | 224 (85.5) | | | 233 (86.0) | | | 218 (85.2) | | | 230 (83.3) | | | 278 (86.3) | | | 310 (85.6) | | | 136 (85.5) | | | 0.94 |  |  |
| Uninsured / self-pay | 92 (35.1) | | | 122 (45.0) | | | 109 (42.6) | | | 112 (40.6) | | | 165 (51.2) | | | 179 (49.5) | | | 74 (46.5) | | | **<0.001** |  |  |
| NH white | 145 (55.3) | | | 163 (60.2) | | | 133 (52.0) | | | 127 (46.0) | | | 147 (45.7) | | | 162 (44.8) | | | 65 (40.9) | | | **<0.001** |  |  |
| Hispanic | 60 (22.9) | | | 60 (22.1) | | | 51 (19.9) | | | 50 (18.1) | | | 71 (22.1) | | | 65 (18.0) | | | 38 (23.9) | | | 0.49 |  |  |
| NH black | 43 (16.4) | | | 35 (12.9) | | | 57 (22.3) | | | 83 (30.1) | | | 87 (27.0) | | | 111 (30.7) | | | 47 (29.6) | | | **<0.001** |  |  |
| NH AAPI/AIAN | 4 (1.5) | | | 3 (1.1) | | | 4 (1.6) | | | 4 (1.5) | | | 7 (2.2) | | | 3 (0.8) | | | 5 (3.1) | | | 0.50 |  |  |
| NH other | 10 (3.8) | | | 10 (3.7) | | | 11 (4.3) | | | 12 (4.4) | | | 10 (3.1) | | | 21 (5.8) | | | 4 (2.5) | | | 0.74 |  |  |
| *Firearm intent* |  | | |  | | |  | | |  | | |  | | |  | | |  | | |  |  |  |
| Assault | 126 (49.1) | | | 136 (50.2) | | | 120 (46.9) | | | 103 (37.3) | | | 145 (45.0) | | | 176 (48.6) | | | 84 (52.8) | | | 0.86 |  |  |
| Unintentional | 62 (24.3) | | | 60 (22.1) | | | 64 (25.0) | | | 75 (27.2) | | | 72 (22.4) | | | 79 (21.8) | | | 31 (19.5) | | | 0.35 |  |  |
| Legal intervention | 6 (2.3) | | | 4 (1.5) | | | 7 (2.7) | | | 11 (4.0) | | | 8 (2.5) | | | 12 (3.3) | | | 4 (2.5) | | | 0.35 |  |  |
| Self-harm | 49 (19.2) | | | 49 (18.1) | | | 38 (14.8) | | | 40 (14.5) | | | 44 (13.7) | | | 44 (12.2) | | | 19 (12.0) | | | **0.004** |  |  |
| Undetermined | 12 (4.7) | | | 22 (8.1) | | | 27 (10.5) | | | 47 (17.0) | | | 53 (16.5) | | | 51 (14.1) | | | 21 (13.2) | | | **<0.001** |  |  |
| *Severity / resource utilization* | | | | | | | | | | | | | | | | | | | | | | | | |
| Severe injury, ISS ≥16 | | 72 (27.5) | | | | 72 (26.6) | | | 68 (26.6) | | | 95 (34.4) | | | 109 (33.9) | | 105 (29.0) | | | 53 (33.3) | | 0.07 | | |
| ICU admission | | 123 (46.9) | | | | 131 (48.3) | | | 120 (46.9) | | | 133 (48.2) | | | 135 (41.9) | | 148 (40.9) | | | 57 (35.9) | | **0.003** | | |
| Hospital LOS, days | | 3 (1-6) | | | | 3 (1-6) | | | 2 (1-6) | | | 3 (1-8) | | | 2 (1-5) | | 2 (1-6) | | | 2 (1-5) | | 0.21 | | |
| ICU LOS, days | | 3 (2-5) | | | | 3 (1-6) | | | 2 (2-3) | | | 3 (2-7) | | | 3 (2-5) | | 3 (2-5) | | | 3 (2-7) | | **0.04** | | |
| Death | | 43 (16.4) | | | | 34 (12.6) | | | 35 (13.7) | | | 46 (16.7) | | | 49 (15.2) | | 48 (13.3) | | | 28 (17.6) | | 0.84 | | |
| Respiratory ventilation | | | | 67 (25.7) | | 65 (24.0) | | | 50 (19.5) | | | 86 (31.2) | | | 95 (29.7) | | 82 (22.7) | | 40 (25.2) | | | 0.71 | | |
| Blood transfusion | | | | 57 (21.8) | | 37 (13.7) | | | 47 (18.4) | | | 71 (25.7) | | | 78 (24.4) | | 67 (18.5) | | 30 (18.9) | | | 0.51 | | |

Data are presented as n (%) or median (interquartile range). Bolding denotes significance, p < 0.05. Abbreviations: H1, first half (1/1/2022-6/30/2022); NH, non-Hispanic; AAPI/AIAN, Asian American Pacific Islander; AIAN, American Indian Alaska Native; ISS, injury severity score; ICU, intensive care unit; LOS, length of stay. ICU LOS calculated for those admitted to the ICU.
